# The effects of elevated pain inhibition on endurance exercise performance

**DOI:** 10.7717/peerj.3028

**Published:** 2017-03-02

**Authors:** Andrew Flood, Gordon Waddington, Richard J. Keegan, Kevin G. Thompson, Stuart Cathcart

**Affiliations:** 1 Research Institute for Sport & Exercise, University of Canberra, Bruce, Australian Capital Territory, Australia; 2 Centre for Applied Psychology, University of Canberra, Bruce, Australian Capital Territory, Australia

**Keywords:** HD-tDCS, CPM, Exercise performance, Pain inhibition

## Abstract

**Background:**

The ergogenic effects of analgesic substances suggest that pain perception is an important regulator of work-rate during fatiguing exercise. Recent research has shown that endogenous inhibitory responses, which act to attenuate nociceptive input and reduce perceived pain, can be increased following transcranial direct current stimulation of the hand motor cortex. Using high-definition transcranial direct current stimulation (HD-tDCS; 2 mA, 20 min), the current study aimed to examine the effects of elevated pain inhibitory capacity on endurance exercise performance. It was hypothesised that HD-tDCS would enhance the efficiency of the endogenous pain inhibitory response and improve endurance exercise performance.

**Methods:**

Twelve healthy males between 18 and 40 years of age (*M* = 24.42 ± 3.85) were recruited for participation. Endogenous pain inhibitory capacity and exercise performance were assessed before and after both active and sham (placebo) stimulation. The conditioned pain modulation protocol was used for the measurement of pain inhibition. Exercise performance assessment consisted of both maximal voluntary contraction (MVC) and submaximal muscular endurance performance trials using isometric contractions of the non-dominant leg extensors.

**Results:**

Active HD-tDCS (pre-tDCS, −.32 ± 1.33 kg; post-tDCS, −1.23 ± 1.21 kg) significantly increased pain inhibitory responses relative to the effects of sham HD-tDCS (pre-tDCS, −.91 ± .92 kg; post-tDCS, −.26 ± .92 kg; *p* = .046). Irrespective of condition, peak MVC force and muscular endurance was reduced from pre- to post-stimulation. HD-tDCS did not significantly influence this reduction in maximal force (active: pre-tDCS, 264.89 ± 66.87 Nm; post-tDCS, 236.33 ± 66.51 Nm; sham: pre-tDCS, 249.25 ± 88.56 Nm; post-tDCS, 239.63 ± 67.53 Nm) or muscular endurance (active: pre-tDCS, 104.65 ± 42.36 s; post-tDCS, 93.07 ± 33.73 s; sham: pre-tDCS, 123.42 ± 72.48 s; post-tDCS, 100.27 ± 44.25 s).

**Discussion:**

Despite increasing pain inhibitory capacity relative to sham stimulation, active HD-tDCS did not significantly elevate maximal force production or muscular endurance. These findings question the role of endogenous pain inhibitory networks in the regulation of exercise performance.

## Introduction

Models of exercise intensity regulation, such as the central governor model (CGM), argue that work-rate during the performance of fatiguing exercise tasks is regulated by a centrally derived pacing strategy (St Clair Gibson & Noakes, 2004). The central integration of afferent signals from the periphery is thought to be involved in the calculation of this pacing strategy (Lambert, St Clair Gibson & Noakes, 2005). An alternative model, the psychobiological model, also suggests that exercise intensity is centrally regulated (Marcora, 2008). However, according to this model, the regulation of work-rate is dependent on the conscious perception of effort and the maximum perception of effort that the individual is willing to withstand (Marcora, Bosio & De Morree, 2008). As perceived effort is considered to be independent of afferent feedback, the psychobiological model argues that exercise intensity regulation occurs without the involvement of sensory feedback from the periphery (Marcora, 2010).

In an attempt to clarify the role of afferent feedback, research has investigated the effects of experimentally manipulating nociceptive afferents and perceived pain on exercise performance. Acetaminophen, a common analgesic, has been shown to increase cycling performance (Mauger, Jones & Williams, 2010). Similarly, the ergogenic effects of caffeine consumption have been attributed to the substance’s analgesic properties (Gonglach et al., 2015). Naloxone, an opioid antagonist, was shown to decrease time-to-exhaustion in an incremental cycling task (Sgherza et al., 2002). Combined, these research findings offer support for the role of afferent feedback in exercise intensity regulation proposed by the CGM.

Despite this evidence pointing to an important role for pain in the regulation of endurance exercise, an examination of the endogenous processes involved in the central modulation of nociceptive input is lacking. The perception of pain is dependent on the activity of central endogenous modulatory processing, involving inhibitory and facilitatory mechanisms (Millan, 2002; Nir & Yarnitsky, 2015). Endogenous pain inhibition acts by inhibiting nociceptive input in the central nervous system at both spinal and supraspinal levels, with descending neural pathways with inputs from cortical, subcortical and spinal regions thought to be involved in this modulatory process (Ossipov, Dussor & Porreca, 2010). Activation of inhibitory pathways has been shown to produce strong, widespread analgesia (Le Bars, Dickenson & Besson, 1979). Importantly, nociceptive afferents originating in the muscle also undergo extensive endogenous modulation which acts to enhance or diminish the intensity of exercise-induced pain (O’Connor & Cook, 1999).

It is possible that a highly efficient endogenous pain inhibitory system is adaptive for endurance exercise performance. In support of this hypothesis, it has previously been proposed that exogenous interventions which enhance the efficiency of the endogenous pain modulatory system may produce an exercise performance advantage (Angius et al., 2015; Mauger, Jones & Williams, 2010). However, previous research in this field has been limited by the exclusive use of static measures of pain assessment, such as tolerance and threshold. Advances in pain psychophysics have led to the development of dynamic pain assessment tools which, unlike static measures, allow for the measurement of the efficiency of the endogenous pain modulatory system (Yarnitsky, 2010). Of these assessment tools, conditioned pain modulation (CPM) is the method typically used to assess endogenous pain inhibition. The ability to measure endogenous pain inhibitory capacity offers an opportunity to examine the speculated role of endogenous pain inhibition in exercise performance.

Using the CPM assessment protocol, Flood, Waddington & Cathcart (in press) recently found a significant relationship between endogenous pain modulation and endurance exercise performance, with those displaying more efficient endogenous pain modulatory responses capable of producing longer endurance times in a submaximal isometric contraction of the knee extensors. Despite these observational findings suggesting a relationship between endogenous pain modulation and endurance exercise performance, it remains unclear whether a more efficient endogenous pain modulatory system results in superior endurance exercise performance. To allow for causal conclusions to be drawn, an experimental manipulation of the endogenous pain modulatory system is required.

Transcranial Direct Current Stimulation (tDCS) presents as a tool which may allow for the manipulation of the endogenous pain modulatory system. tDCS involves the application of a low-intensity electrical current to a targeted cortical region, resulting in polarity dependent shifts in the excitability of underlying regions (Nitsche & Paulus, 2000). These neuronal changes have been shown to persist up to 2 h beyond the stimulation period (Kuo et al., 2013). Conventional methods of tDCS involve the application of a direct current through two, typically 35 cm^2^, rectangular sponge electrodes. This results in dispersed current flow beyond the targeted region, reducing the clarity of the behavioural observations (Datta et al., 2009). However, computational modelling has shown that an advanced method of tDCS, high-definition tDCS (HD-tDCS), allows for far more focal stimulation, restricting current flow to the targeted region (Kuo et al., 2013).

Recent research has shown that conventional tDCS (Reidler et al., 2012) as well as the more focal HD-tDCS (Flood, Waddington & Cathcart, 2016) can enhance the efficiency of the endogenous pain modulatory system, causing transient increases in the endogenous pain inhibitory responses of healthy participants, as assessed through the CPM protocol. Through cortico-cortico and cortico-subcortical projections, it is thought that tDCS targeted over the hand area of the motor cortex stimulates descending regions associated with endogenous pain inhibition, enhancing central pain inhibitory responses and causing widespread analgesia (Flood, Waddington & Cathcart, 2016). Therefore, by acting upon regions involved in the endogenous pain modulatory system, tDCS presents as a viable tool for the top-down inhibition of ascending exercise-induced nociceptive input.

Using HD-tDCS, the current study aimed to manipulate the efficiency of the endogenous pain modulatory system to assess the influence of enhanced endogenous pain inhibitory capacity on endurance exercise performance. It was hypothesised that HD-tDCS of the hand motor cortex would result in an elevated endogenous pain inhibitory response and increased endurance exercise performance.

## Methods

### Participants

To control for potential gender (Popescu et al., 2010) and age (Grashorn et al., 2013) related variations in pain inhibitory capacity, 12 healthy, recreationally active males between 18 and 40 years of age were recruited for participation. All participants were recruited from the local University student population. Participants were not engaged in regular strength training programs or competitive sports predominantly involving knee extensor muscles (e.g., cycling, weightlifting). They were also free from symptoms of chronic pain and did not present with contraindications to tDCS (see Villamar et al., 2013).

Participants were instructed to refrain from consuming caffeine or analgesic medications or engaging in vigorous physical activity for at least 24 h prior to the experimental sessions. Adherence to these instructions was confirmed on arrival.

In Cogiamanian et al. (2007), changes in endurance performance following tDCS were assessed across active anodal (*n* = 9; *M* =  − 21.11 ± 5.52), active cathodal (*n* = 9; *M* =  − 35.77 ± 3.39) and control (*n* = 15; *M* =  − 39.33 ± 3.32) conditions. A-priori power analysis based on data presented in Cogiamanian et al. (2007) indicated a total sample of eight participants was needed to uncover a statistically significant difference in endurance time between active and sham conditions with a statistical power of 0.80 at an alpha level of 0.05.

### Pain inhibition assessment

In the current study, pain inhibitory capacity was assessed using a sequential CPM design, whereby a noxious test stimulus (TS) is presented before and immediately after a noxious conditioning stimulus (CS) is applied to a spatially remote bodily region. This method is in contrast to the simultaneous or parallel CPM design where the second TS and the CS are presented simultaneously. The sequential design was preferred as it has been suggested to provide a clearer indication of endogenous pain modulation (Yarnitsky et al., 2015).

Pressure pain threshold (PPT) assessment was used as the TS. Using a manual pressure algometer (Wagner Force Dial FDK 20, Wagner Instruments, Greenwich, CT), pressure was gradually applied (approximately 1 kg/s) to the dorsal surface of the index finger of participants’ non-dominant hand. Participants were instructed to verbally indicate when the pressure first caused pain, at which point the pressure was released and the peak force required to elicit pain was recorded. After 30 s, this assessment was repeated, allowing for an average of the two trials to be calculated. Participants wore a blindfold throughout PPT assessment so as to minimise potential distraction. The use of PPT assessment as the TS in the CPM protocol is well accepted (see Pud, Granovsky & Yarnitsky, 2009).

Cuff occlusion of the non-dominant arm was used as the CS. A sphygmomanometer cuff was placed approximately 2 cm proximal to the cubital fossa and gradually inflated (∼20 mmHg/s) until participants first reported pain. The cuff then remained at this level of inflation while participants verbally rated their perceived pain level at 10 s intervals using a 0–10 numerical rating scale (0–10_NRS_). If reported pain remained stable for two consecutive intervals, or if ratings of perceived pain decreased, then the cuff was inflated by ∼20 mmHg. Once a perceived pain intensity of *5* on the 0–10_NRS_ was reported, the cuff was fully deflated. Cuff occlusion has previously been shown to be an effective CS for the assessment of pain inhibitory capacity (Cathcart et al., 2009).

Immediately following the removal of the CS, PPT was assessed again. As recommended by Yarnitsky et al. (2015), CPM was calculated by subtracting post-conditioning PPT from pre-conditioning PPT. Therefore, lower CPM values represent enhanced pain inhibitory capacity.

### Force measurement for exercise performance trials

An isokinetic dynamometer (Humac NORM, Computer Sports Medicine, Stoughton, MA) was used to assess force production during maximal voluntary contraction (MVC) and endurance tasks. Participants sat in an upright position perpendicular to the dynamometer while adjustments were made to ensure the correct alignment of the dynamometer’s axis of rotation with the lateral femoral epicondyle of the non-dominant leg. To limit movement during force production, participants were fastened to the chair at the shoulders and hip as well as the thigh of the involved leg. Throughout both performance tasks, force was applied by the non-dominant leg, fixed at 90° of flexion, through the padded arm of the dynamometer fastened slightly above the medial malleolus.

### Maximal voluntary force production

Three submaximal familiarisation trials were performed 60 s prior to the MVC trials. For MVC assessment, participants were instructed to maximally contract their non-dominant leg for 5 s. Two trials, separated by 60 s, were completed prior to the submaximal fatiguing task. Visual feedback relating to force production and elapsed time was presented on a computer monitor throughout the MVC trials. Scripted verbal encouragement was also provided by the researcher. Peak torque (Nm) from the better of the two trials was taken as the MVC. Pre-tDCS MVC was used to calculate the target force for the fatiguing task.

### Muscular endurance assessment

For the assessment of muscular endurance, a fatiguing task was performed whereby participants were required to maintain a submaximal isometric contraction at or above 30% of their pre-tDCS MVC force for as long as possible. Task failure was defined as an inability to produce force at or above the target for three consecutive seconds. To promote maximal performance while also limiting the potential for experimenter bias, participants received scripted verbal encouragement throughout the task. Participants also received visual feedback on a computer monitor indicating current and target force. No feedback regarding elapsed-time was given to participants during the fatiguing task.

### Direct current stimulation

**Figure 1:**
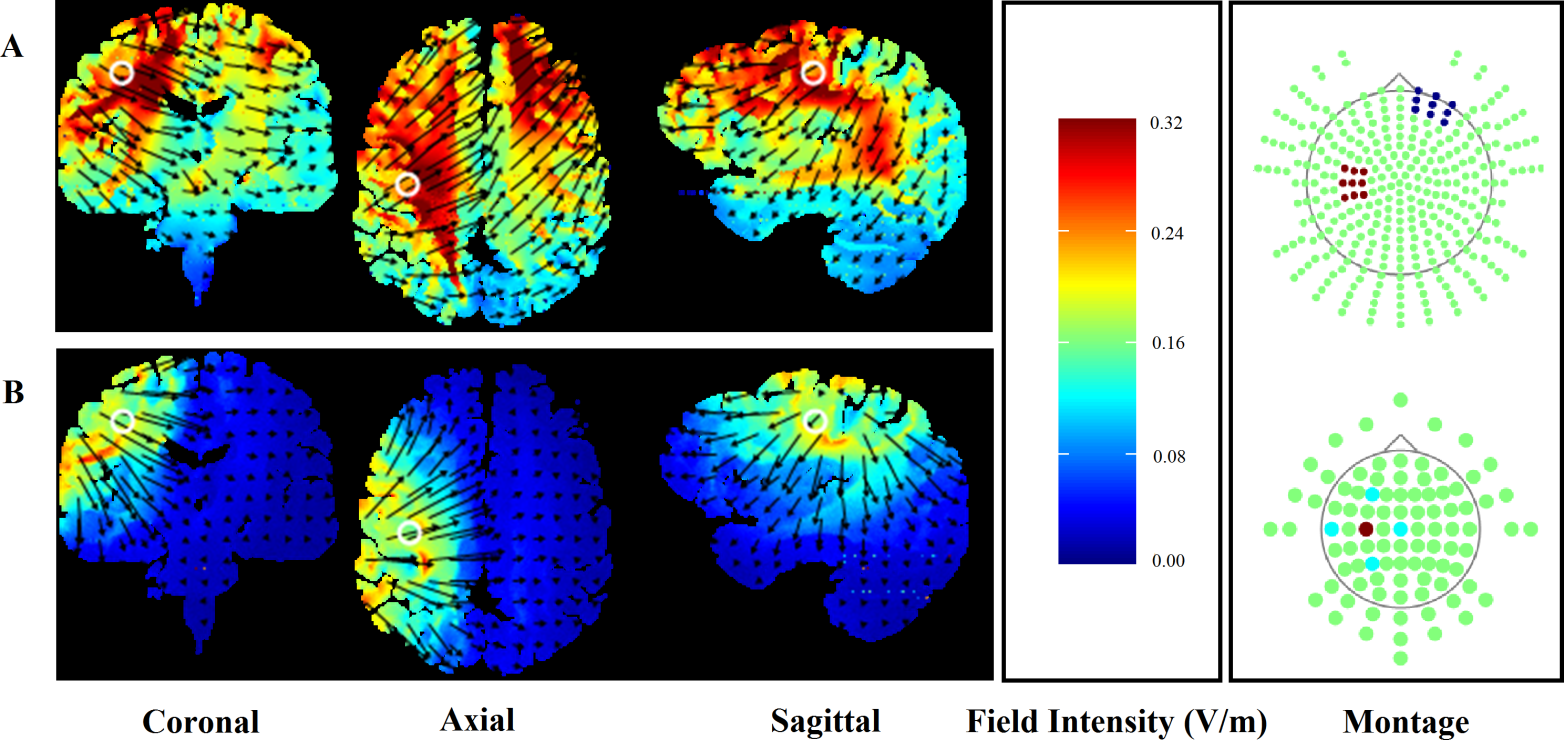
Finite element models of conventional (A) and HD-tDCS (B) of the right hand motor cortex (C3) using HDExplore computational modelling software. Electrode placement is presented with red markers representing anodal and blue markers representing cathodal electrodes. Slice positions are taken at MNI coordinates of −31, −8, and 40 for sagittal, coronal, and axial slices, respectively. A field intensity range of 0.00–0.32 was used in both conventional and HD-tDCS models. White circles represent the centre position of each slice while the direction of the current flow is indicated by black arrows.

To ensure participant safety, the application of HD-tDCS adhered to the procedural recommendations of Villamar et al. (2013). A HD-tDCS multi-channel stimulation interface (Model 4X1-C2, Soterix Medical, New York, NY) attached to a 1 × 1 low-intensity direct current stimulator (Model 1300, Soterix Medical) delivered 20 min of HD-tDCS. Following the International 10/10 electroencephalography system, electrodes were placed in a 4 × 1 ring configuration with the centre electrode positioned on the scalp over the hand motor cortex contralateral to the non-dominant side (C3/C4) and return electrodes positioned in a ring around the centre anode at a radius of approximately 5 cm corresponding to Cz, F3/F4, T7/T8 and P3/P4. Electrodes were held in place using an electroencephalography recording cap. Both electrode placement and stimulation intensity replicated the methods used in previous research showing enhanced CPM following HD-tDCS (Flood, Waddington & Cathcart, 2016). Electrode placement over the hand motor cortex was also chosen to ensure that any functional change in the performance of the leg motor tasks was not due to increased cortico-spinal excitability of the involved limb. HD-Explore software (Version 2.3, Soterix Medical, New York, NY) was used to confirm the focality of the HD-tDCS induced electrical field (Fig. 1). For comparison, computational modelling for the conventional 35 m^2^ sponge-electrodes is also presented (Fig. 1).

Participants received both active and sham (placebo) stimulation conditions. The active condition began with an initial 10 s ramping up period, where stimulation intensity was gradually increased from 0 mA to the target intensity of 2 mA. The current then remained at 2 mA for 20 min, followed by a gradual (10 s) ramping down to 0 mA.

Protocols for the administration of sham stimulation followed those of the active condition. However, in the sham condition, stimulation intensity ramped up to 2 mA and then immediately back down to 0 mA at the start and again at the end of the 20 min period. Previous research has supported the use of similar sham protocols as successful methods for the blinding of participants to their experimental condition (Borckardt et al., 2012). Protocols for the delivery of active and sham stimulation, including ramping periods, were automated by the stimulation device, ensuring consistency throughout the sample.

### Motivation

The scale developed by Matthews, Campbell & Falconer (2001) was used for the assessment of motivation relating to the physical performance tasks. This scale measures success and intrinsic motivation for an upcoming task. All items are scored according to a five-point Likert scale. Higher total scores represent greater levels of motivation for both domains of motivation.

### Procedure

Study protocols followed those approved by the local human research ethics committee (Project: 15-261). Prior to testing, written informed consent was obtained. Participants attended two testing sessions separated by at least 1 wk, where they received active or sham stimulation in a randomised counterbalanced order. A single-blind design was adopted, with participants unaware of the ordering of the stimulation conditions.

**Figure 2:**
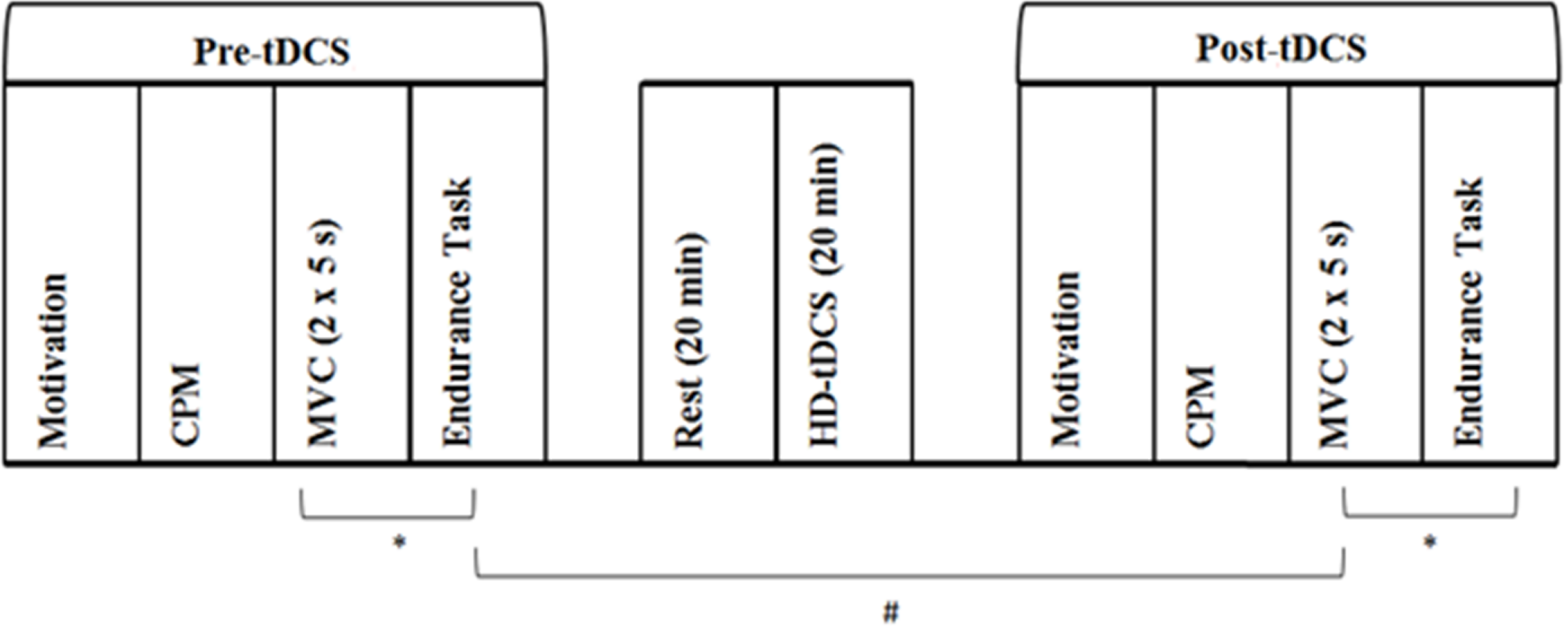
Experimental protocol assessing the effect of increased pain inhibition on exercise performance. First, baseline (Pre-tDCS) measurement of motivation, CPM, MVC and muscular endurance was conducted. The CPM protocol involved PPT assessment before and immediately after cuff occlusion of the opposing upper arm. MVC assessment involved two, 5 s maximal voluntary isometric contractions of the non-dominant leg separated by 60 s. Muscular endurance was assessed as time to failure in a sustained isometric contraction at 30% of peak MVC force. Following a 20 min rest period, 20 min of HD-tDCS at 2 mA was delivered. Motivation, CPM, MVC and muscular endurance were then assessed again (Post-tDCS). A 4 min rest period (∗) separated the MVC and endurance trials. Pre- and post-tDCS physical performance assessment was separated by 1 h (#). Participants attended two sessions which differed in the type of stimulation delivered, with active and sham HD-tDCS administered in a randomised, counterbalanced order. Otherwise, experimental protocols for sessions one and two were identical.

Motivation, pain inhibition, MVC, and muscular endurance were assessed before and after the HD-tDCS intervention. The calculation of the target force for both pre- and post-tDCS muscular endurance trials was based on pre-tDCS MVC performance. Pre- and post-tDCS physical performance measures were separated by 1 h. During this period, participants rested for 20 min, then underwent 20 min of HD-tDCS followed by post-tDCS motivation and CPM assessment. An additional 4 min rest period separated MVC and endurance trials. See Fig. 2 for schematic description of methods.

### Data analysis

Data analysis was performed using SPSS statistical software (Version 21, IBM Corp, Armonk, NY). For all analyses, *p* < .050 was considered statistically significant. Means and standard deviations for all dependent variables are presented in Table 1. In accordance with prescribed publication guidelines (APA, 2010), significance values (*p*-values) and effect sizes (Cohen’s *d* or partial *η*^2^) are presented for all analyses.

Prior to analysis, assumptions for parametric tests were assessed and confirmed. Paired samples *t*-tests were used to assess differences in pre-tDCS motivation, pre-conditioning PPT, CPM, MVC and muscular endurance between the two sessions. Two-way repeated measures ANOVAs examined the effect of anodal and sham stimulation on MVC, muscular endurance, pre-conditioning PPT, CPM and motivation with the main factors of time (pre- and post-tDCS) and condition (active and sham). Normalised values were also calculated for peak MVC force (MVC-norm) and muscular endurance (endurance-norm) as (post-tDCS–pre-tDCS)/pre-tDCS. Therefore, these normalised values represent the relative (percentage) change in performance from pre- to post-tDCS, with negative values indicating a reduction in performance. Paired samples *t*-tests compared MVC-norm and endurance-norm across the two conditions.

**Table 1:** Mean and standard deviations for pre- and post-tDCS motivation, pre-conditioning PPT, CPM, MVC and muscular endurance across active and sham sessions.

	Active		Sham	
	Pre-tDCS	Post-tDCS	Pre-tDCS	Post-tDCS
Intrinsic motivation	24.92 (1.31)	24.48 (1.73)	24.67 (1.83)	23.92 (2.71)
Success motivation	16.08 (4.50)	17.25 (4.09)	15.33 (4.40)	15.33 (3.20)
Pre-conditioning PPT	12.95 (4.63)	12.21 (4.26)	12.25 (4.03)	12.73 (4.83)
CPM	−.32 (1.33)	−1.23 (1.21)	−.91 (.92)	−.26 (.92)
MVC (Nm)	264.89 (66.87)	236.33 (66.51)	249.25 (88.56)	239.63 (67.53)
Et (s)	104.65 (42.36)	93.07 (33.73)	123.42 (72.48)	100.27 (44.25)

**Notes.**

Data is presented as mean (SD).

Pre-conditioning PPT Pressure pain threshold prior to the presentation of the conditioning stimulus CPM Difference between pre- and post-conditioning PPTs MVC Peak torque achieved over the two maximal 5 s contractions Et Time to exhaustion in the fatiguing exercise task

## Results

A total of 12 participants (mean age =24.42 ± 3.85; mean height = 178.50 ± 7.73; mean weight: 81.13 ± 8.61) were included in statistical analysis. Cortical stimulation was well-tolerated by all participants, with no complaints of deleterious responses to the intervention.

### Baseline assessment

Statistical analysis confirmed that pre-tDCS success motivation, *t*(11) = .80, *p* = .441, *d* = .17, intrinsic motivation, *t*(11) = .82, *p* = .429, *d* = .16, pre-conditioning PPT, *t*(11) =  − .63, *p* = .541, *d* = .16, CPM, *t*(11) = 1.07, *p* = .307, *d* = .52, MVC, *t*(11) = 1.68, *p* = .121, *d* = .20 and muscular endurance, *t*(11) =  − 1.75, *p* = .109, *d* = .32 did not differ between the two sessions.

### Motivation

Self-reported success and intrinsic motivation (Table 1) exceeded the levels observed in previous research (Marcora, Staiano & Manning, 2009). Statistical analysis revealed that levels of success motivation did not differ over time, *F*(1, 11) = 1.77, *p* = .211, partial *η*^2^ = .14, or condition, *F*(1, 11) = 2.39, *p* = .150, partial *η*^2^ = .18. Similarly, levels of intrinsic motivation did not differ over time, *F*(1, 11) = 2.93, *p* = .115, *d* = .21, or condition *F*(1, 11) = 2.71, *p* = .128, *d* = .20. There was also no significant time × condition interaction effect for success motivation, *F*(1, 11) = 1.92, *p* = .194, partial *η*^2^ = .15, or intrinsic motivation, *F*(1, 11) = .92, *p* = .358, *d* = .08, indicating that HD-tDCS did not affect participant motivation.

### Pain measures

Pre-conditioning PPTs did not differ significantly as a function of time, *F*(1, 11) = .15, *p* = .708, *d* = .01, or condition, *F*(1, 11) = .02, *p* = .893, *d* = .00. The time × condition interaction effect for pre-conditioning PPT was also non-significant, *F*(1, 11) = 1.06, *p* = .324, *d* = .09.

Although there was no significant main effect of time, *F*(1, 11) = .21, *p* = .654, partial *η*^2^ = .02, or condition, *F*(1, 11) = .31, *p* = .588, partial *η*^2^ = .03, on CPM, a statistically significant interaction effect was uncovered, *F*(1, 11) = 5.07, *p* = .046, partial *η*^2^ = .32, demonstrating increased endogenous pain inhibition in the active condition relative to the sham condition.

### Physical performance

For MVC force, statistical analysis revealed a significant main effect for time, *F*(1, 11) = 9.61, *p* = .010, partial *η*^2^ = .47, but not condition, *F*(1, 11) = .72, *p* = .415, partial *η*^2^ = .06. There was also no significant time × condition interaction for MVC performance, *F*(1, 11) = 2.89, *p* = .117, partial *η*^2^ = .21. In addition, MVC-norm did not differ significantly between active (−11.24 ± 9.42%) and sham (−.31 ± 15.22%) conditions, *t*(11) = 1.91, *p* = .083, *d* =  − .89.

Muscular endurance decreased from pre- to post-tDCS irrespective of condition, *F*(1, 11) = 5.75, *p* = .035, partial *η*^2^ = .34. However, there was no significant main effect for condition, *F*(1, 11) = 2.53, *p* = .140, partial *η*^2^ = .19. The reduction in endurance performance from pre- to post-sham stimulation (*M* = 23.15 ± 37.67 s reduction) was greater than the reduction from pre- to post-active stimulation (*M* = 11.58 ± 15.81 s reduction). When expressed as a relative change from pre- to post-tDCS, endurance time decreased by 13.30 ± 24.56% following sham stimulation, but only 9.39 ± 12.55% following active. However, both the relative, *t*(11) = .68, *p* = .511, *d* = .21, and absolute, *F*(1, 11) = 1.95, *p* = .190, partial *η*^2^ = .15, performance advantages gained from active HD-tDCS were not deemed statistically significant.

## Discussion

Exercise-induced pain is considered an important factor in exercise performance regulation (Mauger, 2014). With exercise-induced pain being subject to endogenous modulation (O’Connor & Cook, 1999), the strength of this endogenous modulatory system may be important for exercise performance. The current study aimed to assess this possibility by examining the effect of enhancing the efficiency of the endogenous pain modulatory system on endurance exercise performance.

The current findings demonstrate that active HD-tDCS applied over the hand motor cortex does enhance the efficiency of the endogenous pain modulatory system relative to the effects of sham stimulation, which is in agreement with previous research (Flood, Waddington & Cathcart, 2016). However, this relative increase in the efficiency of the endogenous pain modulatory system does not result in a concomitant increase in the muscular endurance exercise performance of the non-dominant leg extensors in motivated individuals.

Previous research has speculated upon the potential influence of endogenous pain modulatory capacity on exercise performance. Mauger, Jones & Williams (2010) argue that analgesic substances which produce performance-enhancing effects act by facilitating the endogenous pain modulatory system. In addition, athletes have been shown to possess a more efficient endogenous modulatory response when compared to non-athlete controls (Flood et al., 2016), and a more efficient endogenous pain modulatory system has been shown to be related to superior endurance exercise performance (Flood, Waddington & Cathcart, in press). Together, these findings suggest that an efficient endogenous pain modulatory system is adaptive for endurance exercise performance. However, the findings of the current study refute this speculation and instead suggest that the efficiency of the endogenous pain modulatory system, assessed through the CPM protocol, does not impact on endurance exercise performance.

Several explanations may account for the current novel findings. Theoretical models of endurance performance, such as the CGM, propose a central regulation of performance which integrates multiple sensory afferents, including nociception, to ensure that homoeostasis is protected and a physiological reserve is maintained during fatiguing exercise tasks (Lambert, St Clair Gibson & Noakes, 2005). In contrast, the psychobiological model of endurance performance proposes that afferent signals from the periphery are not involved in the regulation of work-rate during fatiguing exercise (Marcora, 2009). According to this central tenent of the psychobiological model, changes in endogenous pain modulatory capacity should not produce changes in endurance exercise performance. Therefore, the predictions of the psychobiological model offer an explanatory framework through which the current findings may be viewed.

However, caution should be taken when interpreting the current findings as evidence against the proposed role of pain in the regulation of endurance exercise performance. Endogenous inhibition produces extrasegmental modulatory effects at the spinal dorsal horn, where there is significant convergence of nociceptive input with origins in the skin and muscle (Millan, 1999). Therefore, the manipulation of the endogenous pain modulatory system, as shown through the CPM protocol, was expected to produce heightened widespread endogenous hypoalgesia, influencing nociceptive input from the exercising muscles. However, as in-task assessment of muscle pain was not conducted, it is unclear whether the observed relative increase in the endogenous pain inhibitory response induced via active HD-tDCS translated to an increased inhibition of exercise-induced pain. Therefore, the current findings may instead indicate that the endogenous inhibitory networks responsible for the processing of exercise-induced nociceptive input are distinct from the inhibitory networks affected by HD-tDCS of the hand motor cortex. The aetiologically distinct modulatory pathways for the processing of noxious stimuli (mechanical, thermal, etc.) offers indirect support for this possibility (Millan, 2002). Angius et al. (2015) also suggest that aetiologically distinct pathways are responsible for the processing of exercise-induced nociception and noxious thermal stimuli. We encourage additional research into the neurophysiological pathways specifically responsible for the modulatory processing of exercise-induced pain so as to guide future analgesic interventions for performance-enhancement.

Outside of the effects on the endogenous pain modulatory system, previous research has also directly assessed the ergogenic effects of tDCS. This research has uncovered mixed results. Cogiamanian and colleagues (2007) and more recently Abdelmoula, Baudry & Duchateau (2016) applied conventional tDCS at 1.5 mA for 10 min over the hand motor cortex. In both studies, the reduction in time-to-exhaustion from pre- to post-tDCS assessment was significantly reduced in the active anodal condition relative to either sham (Abdelmoula, Baudry & Duchateau, 2016) or control (Cogiamanian et al., 2007) conditions. It is interesting to note that the endurance performance reductions from pre- to post-tDCS in the anodal (21.1% reduction), cathodal (35.7% reduction) and control conditions (39.3%) reported by Cogiamanian et al. (2007) were substantially greater than those observed in the current study (active, 9.39 ± 12.55% reduction; sham, 13.30 ± 24.56% reduction). In addition, rather than the sham condition used in the current study, Cogiamanian et al. (2007) implemented a control condition which involved no placebo stimulation. These differences in the control conditions used and the degree of fatigue carried from pre- to post-tDCS assessment may explain the discrepancy between the two findings. In contrast to the reported ergogenic effects of tDCS, using similar performance and stimulation protocols, Kan, Dundas & Nosaka (2013) reported no improvement in muscular endurance following tDCS. The potential performance enhancing effects of tDCS have also been examined in dynamic, whole-body exercise, with Angius et al. (2015) finding no significant improvements in cycling time to exhaustion following tDCS over the hand motor cortex.

The potential ergogenic effects of motor cortex targeted tDCS have been attributed, in-part, to increased cortico-spinal excitability to the exercising limb (Cogiamanian et al., 2007). In order to manipulate endogenous inhibitory pathways, without influencing cortico-spinal excitability, the current study assessed performance on a leg motor task after HD-tDCS over the hand area of the motor cortex. The lack of an observed performance improvement offers indirect support for the cortico-spinal excitability explanation of the effects of tDCS on exercise performance.

The use of HD-tDCS rather than conventional methods is a novel approach in the field of exercise performance-enhancement. To-date, no research has examined the ergogenic effects of cortical stimulation using HD-tDCS. This method of cortical stimulation allows for a more targeted delivery of direct current, increasing the spatial focality of the current flow and the resulting neural changes (see Fig. 1; Caparelli-Daquer et al., 2012). The exclusive use of conventional sponge-based methods of tDCS delivery limit the conclusions that can be drawn from previous findings, as the current flow and potential cortical regions influenced are widely dispersed (Fig. 1). In addition to the targeting of the hand motor cortex, the use of HD-tDCS rather than conventional tDCS may explain the lack of performance advantage observed in the current study. As shown in Fig. 1, HD-tDCS resulted in significantly reduced current flow to the somatotopical representation of the leg when compared to conventional tDCS. It is possible that this greater specificity accounts for the failure to replicate the performance advantages reported by Cogiamanian et al. (2007). Therefore, although conventional methods may offer the greatest behavioural effects (i.e., enhanced exercise performance), we suggest that future research should aim to utilise the greater spatial specificity allowed through the use of HD-tDCS to better understand the supraspinal factors involved in endurance exercise performance.

### Limitations

Several limitations of the current study should be noted. The endurance performance task required participants to remain focussed on the computer-displayed force trace and ensure that their force production stayed above the target. Within-task pain assessment was not included as the additional cognitive burden on the participant may have produced a sub-maximal performance. Therefore, as discussed above, we cannot say with certainty that the observed relative increase in endogenous pain inhibitory capacity resulting from active HD-tDCS did reduce exercise-induced pain perception. Future research should attempt to address this limitation through the inclusion of pain assessment both at rest and during the performance of the exercise tasks.

It has been widely reported that the performance of physical activity causes acute widespread hypoalgesia (Koltyn, 2000; Koltyn, 2002; Drury et al., 2004). It is possible that pre-tDCS physical performance may have impacted upon post-tDCS pain assessment. However, the exercise-induced hypoalgesic effect decays quickly after the completion of the exercise task (Koltyn, 2000). Therefore, to account for the potential impact of exercise-induced hypoalgesia, pre-tDCS muscular endurance trials and post-tDCS pain assessment were separated by 40 min (Fig. 2). This rest-period was consistent across the two experimental conditions. Statistical analysis confirmed that pain sensitivity returned to resting levels, with pre-conditioning PPTs not differing between pre- and post-tDCS measures.

The spatial specificity of the electrical current applied via HD-tDCS is greater than that of conventional tDCS (Datta et al., 2009). Computational modelling confirmed this assumption for the current montage (Fig. 1). These differences in the distribution of the stimulated neural region resulting from high-definition and conventional tDCS methods limit the potential for comparisons to be made between the current findings and the findings of previous research. In addition, although HD-tDCS allows for greater stimulation specificity, Fig. 1 suggests that cortical stimulation may have dispersed beyond the targeted hand motor cortex, potentially inducing unwanted neuronal changes in neighbouring regions. In particular, cortical stimulation may have induced changes in cortico-spinal excitability to the leg motor region. However, previous research has achieved somatotopically specific effects using conventional tDCS (Tanaka et al., 2009), suggesting that the more targeted form of cortical stimulation utilised in the current study was sufficiently localised to the target region. To further clarify the effects of targeted cortical stimulation on motor function, we encourage the examination of tDCS-induced changes in cortico-spinal excitability using methods such as transcranial magnetic stimulation.

Caution should be taken when generalising the current findings to the wider population. The current study sampled only young, pain-free males. Research suggests that pain inhibitory capacity is reduced with age (Grashorn et al., 2013), and that males possess increased pain inhibitory responses (Popescu et al., 2010). In addition, chronic pain sufferers exhibit maladaptive pain inhibition (Lautenbacher & Rollman, 1997). It is possible then that in these populations, the effects of HD-tDCS on pain inhibition and exercise performance may be different to the effects seen in the current study.

Similarly, task duration is an important factor to consider in the generalisation of the current results. The mean endurance time across all conditions was approximately 100 s. The factors influencing performance regulation are argued to be task-specific, particularly dependent on the duration of the task (Abbiss & Laursen, 2008). It is possible then that the role of endogenous pain inhibition may be more pronounced in longer duration tasks. Indeed, increased cycling performance following acetaminophen ingestion occurred in endurance tasks exceeding 20 min (Mauger, Jones & Williams, 2010). Future research should continue to examine the potential mediating role of task duration on the factors regulating exercise performance.

No habituation trials were conducted prior to the experimental sessions to ensure a consistency in endurance performance prior to the intervention. The novelty of the performance task may have resulted in the natural performance variations diluting the performance effects of the HD-tDCS intervention. However, statistical analysis demonstrated no significant difference in pre-tDCS endurance time or pre-tDCS MVC force over the two sessions. This suggests that the examination of the performance effects of HD-tDCS were conducted from a stable baseline muscular endurance and MVC assessment.

The current study adopted a single blinded experimental design. In order to minimise the potential for experimenter bias, standardised protocols were implemented for the measurement of both pain and exercise performance. For PPT assessment, rate of pressure application (1 kg/s) and the inter-stimulus interval (30 s) remained consistent for all participants. The rate of cuff inflation for occlusion was also consistent (∼20 mmHg/s). To limit the potential for experimenter bias in the exercise performance tasks, performance feedback displayed on a computer monitor was consistent across sessions. Scripted verbal encouragement during the MVC and endurance tasks also accounted for potential experimenter bias.

There is currently a lack of consistency in the CPM methods used for the assessment of pain modulation capacity (Yarnitsky et al., 2015). PPT assessment as the TS in the CPM protocol is widely utilised, however, several alternate methods such as noxious heat are also used (Pud, Granovsky & Yarnitsky, 2009). Methodological inconsistencies are also evident in the methods used for the CS. Following previous research methodologies (Cathcart et al., 2009), cuff occlusion was used to inhibit the perceived intensity of the TS. To ensure that the perceived intensity of the CS was consistent for all participants, the actual intensityand duration of cuff occlusion varied. It is possible that these variations in the actual intensity and duration of the CS produced varied inhibitory responses. Due to these methodological inconsistencies, we urge caution when generalising the magnitude of pain inhibition observed in the current study.

## Conclusion

The current study found that although HD-tDCS allows for the experimental manipulation of the efficiency of the endogenous pain modulatory system, a more efficient endogenous pain modulatory system does not result in improved endurance exercise performance. This novel finding opposes the argument that a more efficient endogenous pain modulatory system is adaptive for endurance exercise performance. We encourage future research to continue to utilise novel methods of brain stimulation, such as HD-tDCS, to examine the factors involved in exercise performance regulation.

##  Supplemental Information

10.7717/peerj.3028/supp-1 Data S1 Dataset

## Additional Information and Declarations

### Data Availability

The following information was supplied regarding data availability: The raw data has been supplied as Data S1.
